# Randomized controlled trial: Standard versus supplemental bowel preparation in patients with Bristol stool form 1 and 2

**DOI:** 10.1371/journal.pone.0171563

**Published:** 2017-02-27

**Authors:** Yueyue Li, Xinyong Jia, Baozhen Liu, Yanmei Qi, Xiubin Zhang, Rui Ji, Yanbo Yu, Xiuli Zuo, Yanqing Li

**Affiliations:** 1 Department of Gastroenterology, Laboratory of Translational Gastroenterology, Qilu Hospital of Shandong University, Jinan, China; 2 Department of Endoscopy, Qianfoshan Hospital of Shandong University, Jinan, China; 3 Department of Gastroenterology, Binzhou People’s Hospital, Binzhou, China; University Hospital Llandough, UNITED KINGDOM

## Abstract

**Background:**

Bristol stool form 1 and 2 is an important predictor of inadequate bowel preparation.

**Aim:**

To evaluate the efficacy of supplemental preparation in bowel cleansing quality among patients with Bristol stool form 1 and 2, as well as the feasibility of tailored bowel preparation guided by Bristol stool form scale.

**Methods:**

Patients with Bristol stool form 1 and 2 from 3 Chinese tertiary hospitals randomly received either 2 L PEG-ELP (group A) or 10 mg bisacodyl plus 2 L PEG-ELP (group B); patients with Bristol stool form 3 to 7 received 2 L PEG-ELP (group C) for bowel preparation. The primary endpoint is the rate of adequate bowel reparation for the whole colon. The adequate bowel preparation rate for separate colon segments, the polyp detection rate (PDR), tolerability, acceptability, sleeping quality and compliance were evaluated as secondary endpoints.

**Results:**

700 patients were randomized. In per-protocol analysis, patients in group B attained significantly higher successful preparation rate than group A (88.7% vs. 61.2%, p<0.001) and similar with group C (88.7% vs. 85.0%, p = 0.316). The PDR in group B was significantly higher than group A (43.2% vs. 25.7%, p<0.001). Acceptability was much higher in group B and C.

**Conclusions:**

10 mg bisacodyl plus 2 L PEG-ELP can significantly improve both bowel preparation quality and PDR in patients with Bristol stool form 1 and 2. Bristol stool form scale may be an easy and efficient guide for tailored bowel preparation before colonoscopy.

## Introduction

Colonoscopy is the standard approach for evaluating the entire colon currently. Inadequate bowel preparation can result in failed detection of prevalent neoplastic lesions and has been linked to an increased risk of procedural adverse events, lower adenoma detection rates (ADRs), longer procedural time, lower caecal intubation rates, shorter intervals between examinations and an estimated 12–22% increase in overall colonoscopy cost [1–4]. Unfortunately, despite advances in bowel preparation methods [5], it is reported that up to one-third of bowel preparations are inadequate [6–9].

The Bristol stool form scale (BSFS), developed and validated by Kenneth W. Heaton *et al*, has been widely applied in both clinical practice and research [10–12]. According to the shape and consistency, BSFS divides human stool into 7 different types. Each type of stool is sketched with corresponding description and it facilitates patients to ascertain type of their feces [13].

In clinical practice, Bristol stool form is easy to be identified and can predict the quality of bowel preparation [14]. Studies have demonstrated that Bristol stool form 1 and 2 is an important predictor of inadequate bowel preparation [15]. It is recommended that more aggressive bowel preparation regimen, such as 4 L polyethylene glycol (PEG) or low volume preparation plus adjunctive agents, should be prescribed to patients with predictors of inadequate preparation [16]. However, those recommendations are lack of proofs based on randomized controlled studies. What is important, there is no proof-based bowel preparation policy guided by risk factors. BSFS guided bowel preparation is hoped to be easy and efficient in clinical practice.

Bisacodyl is commonly used as the adjunct in bowel preparation. Several studies have demonstrated that bowel preparation quality is similar between regimen of bisacodyl plus 2 L PEG and regimen of 4 L PEG [17, 18].

In this study, we aimed to evaluate the efficacy of supplemental preparation, bisacodyl plus 2 L polyethylene glycol electrolytes powder (PEG-ELP) in bowel cleansing quality among patients with Bristol stool form 1 and 2, as well as the feasibility of tailored bowel preparation guided by Bristol stool form scale.

## Patients and methods

### General

This was a prospective, investigator -blinded, randomized, controlled study with consecutive outpatients undergoing afternoon colonoscopy at three tertiary hospitals in Jinan city and Binzhou city, Shandong province. The study protocol and informed consent form were approved by review boards of the ethic committee of Shandong University Qilu Hospital, the ethic committee of Shandong University Qianfoshan Hospital and the ethic committee of Binzhou People’s Hospital.

The study was registered at www.clinicaltrials.gov (NCT02415569).

### Patients

Outpatients aged 18 or older, undergoing colonoscopy were eligible to participate. Exclusion criteria were: (1) history of colorectal surgery; (2) known or suspected bowel obstruction or perforation; (3) inflammatory bowel disease; (4) severe congestive heart failure (New York Heart Association class III or IV); (5) severe chronic renal failure (creatinine clearance<30 ml/min); (6) pregnancy or lactation; and (7) unable to give informed consent.

### Randomization and masking

At the beginning of entering, BSFS chart with seven corresponding images and descriptions were shown to patients. Each patient reported the main stool form he/she defecated in last 7 days according to the BSFS chart. At the time of appointment for colonoscopy, patients with Bristol stool form 1 and 2 were randomized into either group A or group B by opening a sealed opaque envelope. The envelopes were randomized and blocked by using computer-generated random numbers created by an investigator not involved in the colonoscopy procedure. The competitive enrollment was used in this study. Each center was assigned 1 block (n = 50) for group A and B, and 25 patients for group C at first. When every two patients were enrolled into either group A or group B, the next patient with Bristol stool form 3 to 7 was assigned to group C. Written informed consents were obtained from all the patients. Colonoscopists, nurses and investigators were all blinded to patients’ preparation methods before, during and after the procedures. Study procedures were performed by 5 experienced colonoscopists.

### Preparation regimens

Patients were instructed to take low-residue diet the day before colonoscopy and keep fasting the day of colonoscopy. Patients assigned to group A and C were asked to drink one sachet of PEG-ELP (Polyethylene glycol electrolytes powder (Ⅱ), WanHe Pharmaceutical Co., Shenzhen, China) dissolved in 2 L of water (250 ml every 15 min) .1L was taken 7 h before colonoscopy and another 1L was taken 4h before colonoscopy. Patients in group B were asked to take 2 tablets (10 mg) bisacodyl (Bisacodyl enteric-coated tablet, China Pharmaceutical University Pharmaceutical Co., Nanjing, China) at 8:00 PM the day before the colonoscopy and take 2 L PEG-ELP the same as patients in group A and C. All colonoscopies were performed at 1:30–5:00 PM. A pamphlet printed instructions and cautions of bowel preparation were given to each patient. Additionally, we offered a telephone number to patients, and they were encouraged to dial it if they had any questions about bowel preparation.

### Data collection and colonoscopy

Baseline demographic and clinical characteristics of all patients were recorded at the time of appointment for colonoscopy. On the day of colonoscopy, before their scheduled procedures, patients were interviewed by one investigator who was not involved in the endoscopic procedure in each center. Patients completed a questionnaire evaluating tolerability, acceptability, sleeping quality and compliance. Tolerability was evaluated by the occurrence of adverse events. Acceptability was measured by 3 parameters: satisfaction scores, ease of taking scores and rate of willingness to repeat the same preparation. Satisfaction scores and ease of taking scores were evaluated by using an 8-point Likert scale and ranked from 0 (totally satisfied or extremely easy) to 7 (totally dissatisfied or extremely hard). Sleeping quality was accessed as excellent, good, fair or bad. The bowel preparation time, the food type and the amount of solution intake were recorded to evaluate the compliance.

In each center, one investigator, who was blinded to all information about bowel preparation, recorded the quality of bowel preparation, endoscopic findings, caecal intubation time and withdraw time for all patients. Before study initiation, the 3 investigators were educated by the Boston Bowel Preparation Scale Educational Program (BBPSEP) online (available at http://www.cori.org/bbps/login.php) and performed a calibration exercise on 30 colonoscopies according to BBPS, to achieve a satisfactory level of consistency in the assessment of bowel preparation quality. When the colonoscopy was finished, the colonoscopist reported whether the patient needed a repeat colonoscopy within 1 year. All procedures were conducted with either sedation or awake, according to patients’ willingness.

### Outcome measures

Blinded investigators evaluated preparation quality of each colon segment (right, transverse, and left colon) by using a 4-point scale (0–3) according to the BBPS [19, 20]. Scores of all segments were added up as the total BBPS scores, ranging from 0 to 9. Inadequate was defined as BBPS score <2 in one or more colon segments. The whole colon preparation quality was divided into 4 grades: excellent (total score 8–9), good (total score 6–7, and each colon segment score ≥2), fair (total score 3–5; or total score 6–7, but one or more colon segment score <2) and poor (0–2). The primary study endpoint was the rate of adequate bowel reparation for the whole colon. Secondary endpoint was the adequate bowel preparation (BBPS score ≥2) rate for separate colon segments. Additional secondary endpoints included polyp detection rate (PDR), patient compliance, sleeping quality, tolerability, and acceptability.

### Statistical analysis

We calculated the sample size assuming a 15% difference in the rate of adequate bowel preparation. In our endoscopic center, the rate of adequate bowel preparation in patients with Bristol stool form 1 and 2 was about 60%. Based on assumptions of α = 0.05 and β = 0.1, we calculated that at least 203 patients in each group were needed to detect a statistically significant difference between group A and group B with a two-tailed. Considering 10% of patients may drop out and achieving a 1:1:1ratio in 3 groups, we estimated that a total of 700 patients would be adequate to detect a significant difference in the primary endpoint.

Intention to treat (ITT) analysis and per-protocol (PP) analysis were used to evaluate the primary endpoint. Continuous variables were expressed as means with standard deviation (SD) and analyzed using one-way ANOVA, SNK-q test and LSD-t test. Categorical variables were analyzed using the Pearson chi-square test. Under R-3.2.3, the p values were adjusted using Bonferroni to control false discovery rate. We performed a multivariate binary logistic regression using variables with a p value of <0.1 at univariate analysis to evaluate factors associated with inadequate bowel preparation. Statistical analysis was performed using SPSS software V.17.0 for Windows. A p value <0.05 was considered statistically significant.

## Results

### Patient characteristics

**Fig 1:**
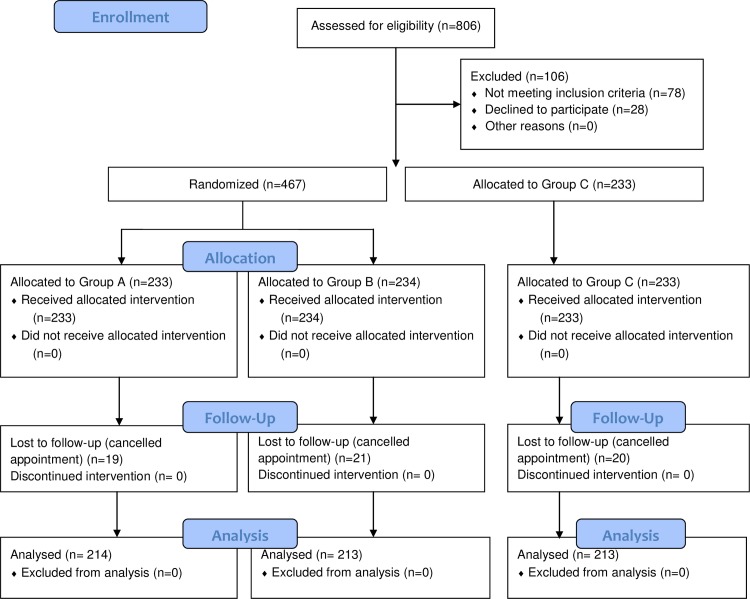
Flowchart of the study. PP, per-protocol.

From January to October 2015, 806 eligible outpatients were assessed for inclusion: 106 were excluded (78 met exclusion criteria and 28 declined to participate in the study); 467 patients with Bristol stool form 1 and 2 were randomized into either group A (n = 233) or group B (n = 234). Patients with Bristol stool type 3–7 were assigned into group C (n = 233). A total of 60 patients cancelled their appointments after randomization, and finally there were 214, 213 and 213 patients going colonoscopy in group A, B and C, respectively (Fig 1). There were no significant differences among 3 groups in baseline characteristics, except that the proportion of female gender in group C is much lower than that in the other two groups (p = 0.001, Table 1).

**Table 1:** Baseline characteristics of the study patients and colonoscopy.

	Group A n = 233	Group B n = 234	Group C n = 233	p Value
Age (years)	52.2±9.7	52.1±9.8	50.2±9.5	0.169
Female gender	156 (67.0%)	160 (68.4%)	123 (52.8%)*	0.001
BMI (kg/m^2^)	23.6±2.5	23.1±2.4	23.4±2.4	0.282
Smoking	35 (15.0%)	37 (15.8%)	43(18.5%)	0.578
Previous surgery (abdominal or pelvic)	42 (18.0%)	47 (20.1%)	39 (16.7%)	0.640
Indication for colonoscopy				
Screening	15 (6.4%)	17 (7.3%)	11 (4.7%)	0.506
Diagnostic	198 (85.0%)	195 (83.3%)	204 (87.6%)	0.431
Surveillance	20 (8.6%)	22 (9.3%)	18 (7.7%)	0.640
Sedation status				0.647
No	152 (65.2%)	143 (61.1%)	146 (62.7%)	
Yes	81 (34.8%)	91 (38.9%)	87 (37.3%)	
Colonoscopy type				0.917
Pentax	177 (76.0%)	174 (74.4%)	176 (75.5%)	
Olympus	56 (24.0%)	60 (25.6%)	57 (24.5%)	

Values are mean±SD, % or number.

BMI, body mass index.

* p values for female gender comparing group A with group B, group A with group C and group B with group C are 0.767, 0.002 and 0.001, separately.

### Bowel preparation quality

Bowel preparation quality was scored and summarized in Table 2. In ITT analysis, the adequate bowel preparation rates of group B and C were both significantly higher than group A (80.8% vs. 56.2%, p_AB_<0.001; 77.7% vs. 56.2%, p_AC_<0.001). There was no significant difference between group B and C in adequate bowel preparation rate (80.8% vs. 77.7%, p_BC_ = 0.427). In PP analysis, both group B and C showed significantly higher adequate bowel preparation rate than group A (88.7% vs. 61.2%, p_AB_<0.001; 85.0% vs. 61.2%, p_AC_<0.001). There was no significant difference between group B and C in adequate bowel preparation rate (88.7% vs. 85.0%, p_BC_ = 0.316).

**Table 2:** Bowel preparation quality in 3 groups in intention to treat (ITT) analysis and per-protocol (PP) analysis.

	Group A	Group B	Group C	p Value				
				p	A-p	p_AB_	p_AC_	p_BC_
ITT population	n = 233	n = 234	n = 233					
Adequate bowel preparation	131(56.2%)	189 (80.8%)	181(77.7%)	<0.001	<0.001	<0.001	<0.001	0.427
PP population	n = 214	n = 213	n = 213					
Adequate bowel preparation	131 (61.2%)	189 (88.7%)	181 (85.0%)	<0.001	<0.001	<0.001	<0.001	0.316
Right colon	141 (65.9%)	193 (90.6%)	187 (87.8%)	<0.001	<0.001	<0.001	<0.001	0.435
Transverse colon	159 (74.3%)	197 (92.5%)	197 (92.5%)	<0.001	<0.001	<0.001	<0.001	1.000
Left colon	184 (86.0%)	203 (94.8%)	202 (94.8%)	<0.001	<0.001	0.001	0.003	1.000
Total BBPS scores	5.6±1.6	7.0±1.1	6.9±1.2	<0.001	<0.001	<0.001	<0.001	0.637
Right colon	1.6±0.6	2.1±0.3	2.0±0.3	<0.001	<0.001	<0.001	<0.001	0.697
Transverse colon	1.8±0.6	2.3±0.6	2.3±0.6	<0.001	<0.001	<0.001	<0.001	0.841
Left colon	2.1±0.6	2.6±0.5	2.5±0.6	<0.001	<0.001	<0.001	<0.001	0.663
Qualitative preparation rating				<0.001	<0.001	<0.001	<0.001	0.558
Excellent	28 (13.1%)	85 (39.9%)	82 (38.5%)					
Good	103 (48.1%)	104 (48.8%)	99 (46.5%)					
Fair	61 (28.5%)	18 (8.5%)	27 (12.7%)					
Poor	22 (10.3%)	6 (2.8%)	5 (2.3%)					

Values are mean±SD, % or number.

ITT, in intention to treat; PP, per-protocol; BBPS, Boston bowel preparation scale.

p_AB_, p value for separate item comparing group A with group B; p_AC_, p value for separate item comparing group A with group C; p_BC_, p value for separate item comparing group B with group C; A-p, adjusted p values. Detailed clinical data is listed in S1 Text.

The adequate preparation rates in right colon were significantly higher in group B and C than that in group A (90.6% vs. 65.9%, p_AB_<0.001; 87.8% vs. 65.9%, p_AC_<0.001). In transverse colon and left colon, the rates showed the similar tendency (p_AB_<0.001, p_AC_<0.001 for transverse colon; p_AB_ = 0.001, p_AC_ = 0.003 for left colon).

Group B and C showed significantly higher total BBPS scores than group A (p_AB_<0.001, p_AC_<0.001). There was no significant difference between group B and C for total BBPS scores (p_BC_ = 0.637). Compared with group A, group B and C showed significantly higher BBPS scores at each colon segment (p<0.001). There was no significant difference between group B and C in terms of BBPS scores at each segment. Patients in group B acquired significantly higher rate of excellent bowel preparation quality than those in group A (39.9% vs. 13.1%, p_AB_<0.001).

### Outcomes of colonoscopy

The outcomes of colonoscopy were shown in Table 3. In group B, successful caecal intubation rate was 94.8%, which was significantly higher than that in group A (88.8%, p_AB_ = 0.033) and similar with that in group C (95.3%, p_AC_ = 0.019), respectively. Incomplete colonoscopy rate due to inadequate preparation was significantly lower in group B than group A (p_AB_ = 0.002). There was lacking of significant difference in caecal intubation time (p = 0.166) and incomplete colonoscopy due to technical difficulty or stricture (p = 0.536) among 3 groups. However, withdrawal time was significantly shorter in group B than group A (p_AB_<0.001). The PDR in group B was significantly higher than that in group A (25.7% vs. 43.2%, p_AB_<0.001) while lacking of significant difference comparing with that in group C (43.2% vs. 37.6%, p_BC_ = 0.277). The rate of need for repeat colonoscopy in 1 year in group A was significantly higher than that in group B and C (p<0.001).

**Table 3:** Outcomes of colonoscopy in 3 groups.

	Group A n = 214	Group B n = 213	Group C n = 213	p Value			
				p	p_AB_	p_AC_	p_BC_
Incomplete colonoscopy	24 (11.2%)	11 (5.2%)	10 (4.7%)	0.013	0.033	0.019	1.000
Inadequate preparation	21 (9.8%)	5 (2.3%)	4 (1.9%)	<0.001	0.002	0.001	1.000
Technical difficulty or stricture	3 (1.4%)	6 (2.8%)	6 (2.8%)	0.536	-	-	-
Successful colonoscopy	190 (88.8%)	202 (94.8%)	203 (95.3%)	0.013	0.033	0.019	1.000
Caecal intubation time (min)	8.6±4.2	7.9±3.6	7.8±3.8	0.166	-	-	-
Withdrawal time (min)	7.5±2.4	6.3±2.1	6.4±1.9	<0.001	<0.001	<0.001	0.900
Polyp detection rate	55 (25.7%)	92 (43.2%)	80 (37.6%)	0.001	<0.001	0.009	0.277
Other colonoscopic findings							
Diverticula	4 (1.9%)	6 (2.8%)	4 (1.9%)	0.768	-	-	-
Colitis	12 (5.6%)	8 (3.8%)	10 (4.7%)	0.664	-	-	-
Cancer	3 (1.4%)	4 (1.9%)	6 (2.8%)	0.574	-	-	-
Other	3 (1.4%)	4 (1.9%)	2 (0.9%)	0.713	-	-	-
Need for repeat colonoscopy within 1 year	44 (20.6%)	13 (6.1%)	17 (8.0%)	<0.001	<0.001	<0.001	0.571

Values are mean±SD, % or number.

p_AB_, p value for separate item comparing group A with group B; p_AC_, p value for separate item comparing group A with group C; p_BC_, p value for separate item comparing group B with group C.

### Tolerability, acceptability, sleeping quality and compliance

The tolerability, acceptability, sleeping quality and compliance are presented in Table 4. The incidence of side effects was not significantly different among 3 groups (p = 0.714). Overall, satisfaction scores in group B and C was significantly lower than that in group A (p_AB_<0.001, p_AC_<0.001). Ease of taking scores of preparation were not significantly different among 3 groups (p = 0.801). The rate of willingness to repeat the same preparation was significantly lower in group A than that in group B (p_AB_ = 0.002) and C (p_AC_ = 0.001). Sleeping quality was not significantly different among 3 groups (p = 0.779). The majority of patients started preparation regimens at correct time (p = 0.938) and abided by correct diet restriction (p = 0.843).

**Table 4:** Other secondary endpoints at per-protocol analysis.

	Group A n = 214	Group B n = 213	Group C n = 213	p Value
Tolerability				
Presence of any of the following side effects, N (%)	72 (33.6%)	76 (35.7%)	68 (31.9%)	0.714
Nausea	50 (23.4%)	48 (22.5%)	51 (23.9%)	0.942
Bloating	24 (11.2%)	30 (14.1%)	24 (11.3%)	0.585
Abdominal pain	10 (4.7%)	16 (7.5%)	11 (5.2%)	0.406
Vomiting	17 (7.9%)	13 (6.1%)	18 (8.5%)	0.713
Lightheadedness	4 (1.9%)	4 (1.9%)	2 (0.9%)	0.668
Acceptability				
Satisfaction scores (0–7 scale)	2.4±1.2	1.9±0.8	1.8±0.8	<0.001*
Ease of taking scores (0–7 scale)	2.1±0.9	2.2±0.9	2.1±1.0	0.801
Willingness to repeat	173 (80.8%)	195 (91.5%)	197 (92.5%)	<0.001#
Sleeping quality				0.779
Excellent or good	185 (86.4%)	179 (84.0%)	182 (85.4%)	
Fair or bad	29 (13.6%)	34 (16.0%)	31 (14.6%)	
Compliance				
Correct start time	197 (92.1%)	195 (91.5%)	197 (92.5%)	0.938
Correct diet restriction	200 (93.5%)	198 (93.0%)	196 (92.0%)	0.843
Amount of solution intake>80%	204 (95.3%)	202 (94.8%)	201 (94.4%)	0.904

Values are mean±SD, % or number.

p_AB_, p value for separate item comparing group A with group B; p_AC_, p value for separate item comparing group A with group C; p_BC_, p value for separate item comparing group B with group C.

* p values for satisfaction scores comparing group A with group B, group A with group C and group B with group C are <0.001, <0.001 and 0.696, separately.

# p values for willingness to repeat comparing group A with group B, group A with group C and group B with group C are 0.002, 0.001 and 0.858, separately.

### Factors associated with inadequate bowel preparation

The significant factors for inadequate bowel preparation were analyzed by logistic regression analysis shown in Table 5. These factors including gender, age, body mass index (BMI), previous surgery (abdominal or pelvic), indication for colonoscopy, diabetes, bowel preparation regimen, patient compliance to product instruction (< 80% intake), any degree of discomfort during preparation, frequency of defecation, defecation times before colonoscopy and Bristol stool form were analyzed. At univariate analysis, factors of inadequate bowel preparation were age>60 years (OR 1.84, p = 0.006), diabetes (OR 3.08, p = 0.001), standard preparation (OR 3.56, p<0.001), poor compliance to product instruction (OR 3.38, p = 0.001), frequency of defecation<3 times/week (OR 1.85, p = 0.002) and Bristol stool form 1–2 (OR 1.80, p = 0.008). Multivariate analysis showed age>60 years (OR 1.63, p = 0.039), diabetes (OR 2.00, p = 0.048), standard preparation (OR 6.81, p<0.001), poor compliance to product instruction (OR 3.67, p = 0.001), frequency of defecation<3 times/week (OR 1.78, p = 0.014) and Bristol stool form 1–2 (OR 2.54, p<0.001) were significant factors for poor bowel preparation.

**Table 5:** Univariate and multivariate analysis of factors associated with inadequate bowel preparation.

	Univariate analysis		Multivariate analysis	
	OR (95% CI)	p value	OR (95% CI)	p value
Age				
≤60 years	Reference		Reference	
>60 years	1.84 (1.21 to 2.79)	0.006	1.63 (1.02 to 2.58)	0.039
Diabetes				
No	Reference		Reference	
Yes	3.08 (1.67 to 5.68)	0.001	2.00 (1.01 to 3.95)	0.048
Standard preparation				
No	Reference		Reference	
Yes	3.56 (2.14 to 5.91)	<0.001	6.81 (3.87 to 11.96)	<0.001
Poor compliance to product instruction				
No	Reference		Reference	
Yes	3.38 (1.66 to 6.90)	0.001	3.67 (1.66 to 8.10)	0.001
Frequency of defecation				
≥3/week	Reference		Reference	
<3/week	1.85 (1.26 to 2.73)	0.006	1.63 (1.02 to 2.58)	0.039
Bristol stool form 1–2				
No	Reference		Reference	
Yes	1.80 (1.16 to 2.78)	0.008	2.54 (1.52 to 4.25)	<0.001

Values are mean±SD, % or number.

OR, odds ratio; CI, confidence interval.

## Discussion

It was found that 10 mg bisacodyl plus 2 L PEG-ELP, performed higher adequate bowel preparation rate and higher BBPS scores in terms of both whole colon and each colon segment than standard regimen in patients with Bristol stool form 1 and 2, and achieved equivalent quality to standard regimen in patients with Bristol stool form 3 to 7 in this study. Previous studies have shown that bisacodyl has the ability to accelerate emptying of ascending colon and colonic transit [21]. It is a commonly used preparation adjunct and has been proved to be effective combining with low volume regimens in many studies [17, 18, 22, 23]. One randomized controlled trial demonstrated that the 2 L of PEG3350e with 15 mg bisacodyl regimen yielded similar bowel preparation quality with 4 L regimen [18]. In a recent randomized, observer-blind study, 400 patients with chronic constipation were randomized into either 2 L PEG plus 15 mg bisacodyl group or 4 L PEG group [23]. The result demonstrated that 2 L PEG plus bisacodyl was similar with 4 L PEG regimen for bowel preparation quality and superior to standard regimen. In this study, we confirmed the ability of bisacodyl plus 2 L PEG-ELP in increasing bowel cleansing quality before colonoscopy.

The PDR in group B could achieved at 43.2%, which was similar with that of 42.0% in Chinese general population [24]. The caecal intubation rate of 94.8% in group B was nearly equivalent to that of 94.9% and higher than that of 92.3% in earlier studies [24, 25]. The intubation and withdraw times in group B were falling in the time frame of previous studies [24, 26]. Inadequate bowel preparation required more suctions and washes in the withdraw procedure, thus more time was needed in group A than other two groups.

Our study showed that 10 mg bisacodyl plus 2 L PEG-ELP did not cause significantly more side effects than standard preparation, consistent with previous studies [18, 27]. According to the current consensus [28], we used 10 mg dose of bisacodyl as the adjunction to 2 L PEG, and we demonstrated this dose was effective and safe. Lower satisfaction in group A is mainly due to the inferior cleansing ability for patients with Bristol stool form 1 and 2. So it is not surprising that the rate of willingness to repeat the same preparation in group A is lower than those in other two groups.

In clinical work, identifying Bristol stool forms of patients is easy and not time-consuming. Our study reveals that it is reasonable to establish one bowel preparation strategy according to BSFS. For this strategy, patients with Bristol stool form 1 and 2 should be prescribed 10 mg bisacodyl plus 2 L PEG-ELP, while patients with Bristol stool form 3 to 7 are prescribed the standard regimen (2 L PEG-ELP). The efficacy of this strategy needs to be validated through multicentre trials with consecutive general patients.

Here, all procedures were performed in the afternoon. Previous researches demonstrated that same-day preparation was either equivalent or superior to split regimen in terms of bowel cleansing quality and was more acceptable for afternoon colonoscopy [29, 30]. Guidelines also recommended the same-day regimen for afternoon procedures [28]. Thus, the same-day regimen was enrolled in this study.

Bristol stool form 1–2 is a risk factor of inadequate bowel preparation in accordance with result of previous study [15]. Poor compliance can strongly predict inadequate bowel preparation both in this study and others [24, 31]. Old age and diabetes were common factors associated with inadequate bowel preparation [9]. Frequency of defecation<3 times/week was demonstrated to be an independent risk factor for inadequate preparation. This can be interpreted that less frequency of defection is associate with slower bowel transit and constipation.

There are some certain limitations in the study. First, instead of the ADR (adenoma detection rate), we only calculated the PDR as previous studies did [22, 23]. We do not routinely to resect or biopsy polyps for outpatients, and thus cannot obtained the pathologic information. Second, we merely involved outpatients into this study. So the efficacy of 10 mg bisacodyl plus 2 L PEG-ELP regimen for Bristol stool form 1 and 2 cannot be verified for inpatients. Third, we merely take Bristol stool form 1and 2 as the guidance in determining bowel preparation regimen in this study. Taking more risk factors, such as old age, diabetes, etc, to guide bowel preparation regimen involvement is hopeful to gain higher adequate bowel preparation rate.

In conclusion, the supplemental preparation, 10 mg bisacodyl plus 2 L PEG-ELP, can significantly improve both total and each colon segment adequate bowel preparation rate. It can also yield better BBPS scores both for whole and separate colon in patients with Bristol stool form 1 and 2. Additionally, the supplemental preparation can significantly improve PDR and acceptability, without impairing tolerability, sleeping quality and compliance in patients with Bristol stool form 1 and 2. Therefore, BSFS guided tailored bowel preparation might be an easy and efficient approach for bowel preparation before colonoscopy.

## Supporting information

S1 CONSORT Checklist CONSORT 2010 Checklist. (DOC)

S1 Study Protocol Study protocol (English version). (DOCX)

S2 Study Protocol Study protocol (Chinese version). (DOCX)

S1 Text Minimal data. (XLSX)
