# Supplementary material for: Randomized controlled trial: Standard versus supplemental bowel preparation in patients with Bristol stool form 1 and 2
Source: PLoS One. 2017 Feb 27;12(2):e0171563. doi: 10.1371/journal.pone.0171563 (PMC5328251; doi:10.1371/journal.pone.0171563)
Supplement: S1 Study Protocol — (DOCX) [file pone.0171563.s002.docx]

**Study protocol**

**Randomized controlled trial: standard versus supplemental bowel preparation in patients with Bristol stool type 1 and 2**

**Introduction**

Colonoscopy is the standard approach for evaluating the entire colon currently. Inadequate bowel preparation can result in failed detection of prevalent neoplastic lesions and has been linked to an increased risk of procedural adverse events, lower adenoma detection rates (ADRs), longer procedural time, lower caecal intubation rates, shorter intervals between examinations and an estimated 12-22% increase in overall colonoscopy cost [1-4]. Unfortunately, despite advances in bowel preparation methods [5], it is reported that up to one-third of bowel preparation were inadequate [6-9].

# The Bristol stool form scale (BSFS), developed and validated by Kenneth W. Heaton *et al*, was widely applied in both clinical practice and research [10-12]. According to its shape and consistency, BSFS divides human stool into 7 different types. Each type of stool was sketched with corresponding description and it facilitates patients to ascertain type of their feces [13].

In clinical practice, Bristol stool form is easy to indentify, and can predict the quality of bowel preparation [14]. Studies have demonstrated that Bristol stool form 1 and 2 is an important predictor of inadequate bowel preparation [15]. It was recommended that more aggressive bowel preparation regimen, such as 4 L PEG or low volume preparation plus adjunctive agent, should be prescribed to patients with predictors of inadequate preparation [16]. However, those recommendations are lack of proofs based on randomized controlled studies. What is important, there is no proof-based bowel preparation policy guided by risk factors. BSFS guided bowel preparation is hoped to be easy and efficient in clinical practice.

Bisacodyl is commonly used as the adjunct in bowel preparation. Several studies have demonstrated that bowel preparation quality is similar between regimen of bisacodyl plus 2 L PEG compared and regimen of 4 L PEG [17, 18].

In this study, we intend to evaluate the efficacy of supplemental preparation, bisacodyl plus 2 L polyethylene glycol electrolytes powder (PEG-ELP) in bowel cleansing quality among patients with Bristol stool form 1 and 2, as well as the feasibility of tailored bowel preparation guided by Bristol stool form scale.

Table 1 The Bristol stool form scale

**Patients and methods**

**General**

This is a prospective, investigator -blinded, randomized, controlled study with consecutive outpatients undergoing afternoon colonoscopy at three tertiary hospitals in Jinan city and Binzhou city, Shandong province. The study protocol and informed consent form has been approved by the Institutional Review Boards before enrolling patients at each center.

The study has been registered at [www.clinicaltrials.gov](http://www.clinicaltrials.gov) (NCT02415569).

**Patients**

Outpatients aged 18 or older, undergoing colonoscopy will be eligible to participate. Exclusion criteria are: (1) history of colorectal surgery; (2) known or suspected bowel obstruction or perforation; (3) inflammatory bowel disease; (4) severe congestive heart failure (New York Heart Association class III or IV); (5) severe chronic renal failure (creatinine clearance<30 ml/min); (6) pregnancy or lactation; and (7) unable to give informed consent.

**Randomization and masking**

At the beginning of entering, BSFS chart with seven types feces images and descriptions will be shown to patients. (Figure 1) Each patient will report the main stool form he/she defecated in last 7 days according to the BSFS chart. At the time of appointment for colonoscopy, patients with Bristol stool form 1 and 2 will be randomized into either group A or group B by opening a sealed opaque envelope. The envelopes are randomized and blocked by using computer-generated random numbers created by an investigator not involved in the colonoscopy procedure.
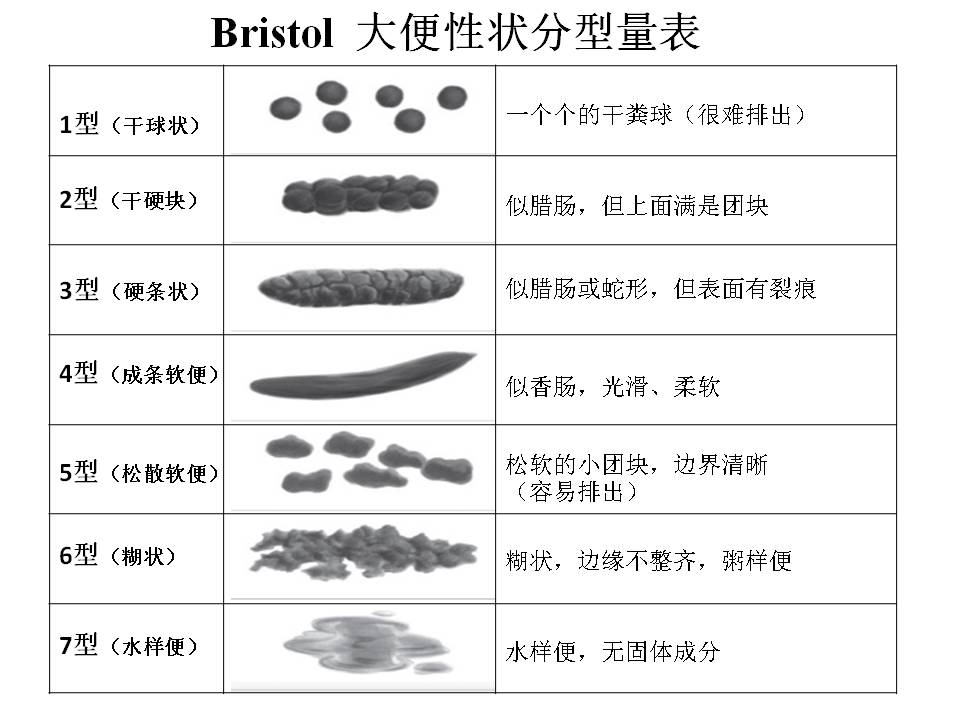


Figure 1 Modified Bristol stool form scale

The competitive enrollment is used in this study. Each center was assigned 1 block (n=50) for group A and B, and 25 patients for group C at first. When every two patients are enrolled into either group A or group B, the next patient with Bristol stool form 3 to 7 is assigned to group C. Written informed consents will be obtained from all the patients. Colonoscopists, nurses and investigators are all blinded to patients’ preparation method before, during and after the procedure. Study procedures will be performed by 5 experienced colonoscopists.

**Preparation regimens**

Patients will be instructed to take low-residue diet the day before colonoscopy and keep fasting the day of colonoscopy. Patients assigned to group A and C are asked to drink one sachet of PEG-ELP (Polyethylene glycol electrolytes powder (Ⅱ), WanHe Pharmaceutical Co., Shenzhen, China) dissolved in 2 liters of water (250 ml every 15 min) .1L is taken 7 h before colonoscopy and another 1L is taken 4h before colonoscopy. Patients in group B will be asked to take 2 tablets (10 mg) bisacodyl (Bisacodyl enteric-coated tablet, China Pharmaceutical University Pharmaceutical Co., Nanjing, China) at 8:00 PM the day before the colonoscopy and take 2 L PEG-ELP the same as patients in group A and C. All colonoscopies were performed at 1:30-5:00 PM. A pamphlet printed instructions and cautions of bowel preparation were given to each patient. Additionally, we will offer a telephone number to patients, and they will be encouraged to dial it if they have any questions about bowel preparation.

**Data collection and colonoscopy**

Baseline demographic and clinical characteristics of all patients will be recorded at the time of appointment for colonoscopy. On the day of colonoscopy, before their scheduled procedure, patients will be interviewed by one investigator in each center who is not involved in the endoscopic procedure. Patients will complete a questionnaire evaluating tolerability, acceptability, sleeping quality and compliance. Tolerability is evaluated by the occurrence of adverse events. Acceptability is measured by 3 parameters: satisfaction scores, ease of taking scores and rate of willingness to repeat the same preparation. Satisfaction scores and ease of taking scores are evaluated by using an 8-point Likert scale and ranked from 0 (totally satisfied or extremely easy) to 7 (totally dissatisfied or extremely hard). Sleeping quality is accessed as excellent, good, fair or bad. The bowel preparation time, the food type and the amount of solution intake are recorded to evaluate the compliance.

In each center, one investigator, who is blinded to all information about bowel preparation, recorded the quality of bowel preparation, endoscopic findings, caecal intubation time and withdraw time for all patients. Before study initiation, the 3 investigators are educated by the Boston Bowel Preparation Scale Educational Program (BBPSEP) online (available at http:// [www.cori.org/bbps/login.php](http://www.cori.org/bbps/login.php)) and performed a calibration exercise on 30 colonoscopies according to BBPS, to achieve a satisfactory level of consistency in the assessment of bowel preparation quality. When the colonoscopy is finished, the colonoscopist reports whether the patient need a repeat colonoscopy within 1 year. All procedures will be conducted with either sedation or awake, according to patients’ willingness.

**Outcome measures**

Blinded investigators will evaluate preparation quality of each colon segment (right, transverse, and left colon) by using a 4-point scale (0-3) according to the BBPS [19, 20]. Scores of all segments are added up as the total BBPS scores, ranging from 0 to 9. Inadequate is defined as BBPS score <2 in one or more colon segments. The whole colon preparation quality is divided into 4 grades: excellent (total score 8-9), good (total score 6-7, and each colon segment score ≥2), fair (total score 3-5; or total score 6-7, but one or more colon segment score <2) and poor (0-2). The primary study endpoint is the rate of adequate bowel reparation for the whole colon. Secondary endpoint is the adequate bowel preparation (BBPS score ≥2) rate for separate colon segments. Additional secondary endpoints included PDR, patient compliance, sleeping quality, tolerability, and acceptability.

**Statistical analysis**

We calculate the sample size assuming a 15% difference in the rate of adequate bowel preparation. In our endoscopic center, the rate of adequate bowel preparation in patients with Bristol stool form 1 and 2 was about 60%. We calculate that at least 203 patients in each group are needed to provide 90% power to detect a statistically significant difference between group A and group B at a two-tailed probability level of 95%. Considering 10% of patients may drop out and achieving a 1:1:1ratio in 3 groups, we estimate that a total of 700 patients would be adequate to detect a significant difference in the primary endpoint.

Intention to treat (ITT) analysis and per-protocol (PP) analysis will be used to evaluate the primary endpoint. Continuous variables will be expressed as means with SD and analyzed using one-way ANOVA, SNK-q test and LSD-t test. Categorical variables are analyzed using the Pearson chi-square test. We performed multivariate analysis using variables with a p value of <0.1 at univariate analysis to evaluate factors associated with inadequate bowel preparation. Statistical analysis will be performed using SPSS software V.17.0 for Windows. A p value <0.05 is considered statistically significant.

**References**

1 Harewood GC, Sharma VK, de Garmo P. Impact of colonoscopy preparation quality on detection of suspected colonic neoplasia. Gastrointest Endosc. 2003;58:76-79.

2 Chokshi RV, Hovis CE, Hollander T, Early DS, Wang JS. Prevalence of missed adenomas in patients with inadequate bowel preparation on screening colonoscopy. Gastrointest Endosc. 2012;75:1197-1203.

3 Rex DK, Imperiale TF, Latinovich DR, Bratcher LL. Impact of bowel preparation on efficiency and cost of colonoscopy. Am J Gastroenterol. 2002;97:1696-1700.

4 Senore C, Ederle A, Fantin A, Andreoni B, Bisanti L, Grazzini G, et al. Acceptability and side-effects of colonoscopy and sigmoidoscopy in a screening setting. J Med Screen. 2011;18:128-134.

5 Connor A, Tolan D, Hughes S, Carr N, Tomson C. Consensus guidelines for the safe prescription and administration of oral bowel-cleansing agents. Gut. 2012;61:1525-1532.

6 Froehlich F, Wietlisbach V, Gonvers JJ, Burnand B, Vader JP. Impact of colonic cleansing on quality and diagnostic yield of colonoscopy: the European Panel of Appropriateness of Gastrointestinal Endoscopy European multicenter study. Gastrointest Endosc. 2005;61:378-384.

7 Chung YW, Han DS, Park KH, Kim KO, Park CH, Hahn T, et al. Patient factors predictive of inadequate bowel preparation using polyethylene glycol: a prospective study in Korea. J Clin Gastroenterol. 2009;43:448-452.

8 Lebwohl B, Wang TC, Neugut AI. Socioeconomic and other predictors of colonoscopy preparation quality. Dig Dis Sci. 2010;55:2014-2020.

9 Hassan C, Fuccio L, Bruno M, Pagano N, Spada C, Carrara S, et al. A predictive model identifies patients most likely to have inadequate bowel preparation for colonoscopy. Clin Gastroenterol Hepatol. 2012;10:501-506.

10 O'Donnell LJD, Virjee J, Heaton KW. Detection of pseudodiarrhoea by simple clinical assessment of intestinal transit rate. BMJ. 1990;300:439-440.

11 K W Heaton, S Ghosh, F E M Braddon. How bad are the symptoms and bowel dysfunction of patients with the irritable bowel syndrome? A prospective, controlled study with emphasis on stool form. Gut. 1991;32:73-79.

12 Lewis SJ, Heaton KW. Stool form scale as a useful guide to intestinal transit time. Scand J Gastroenterol. 1997;32:920-924.

13 Martinez AP, de Azevedo GR. The Bristol Stool Form Scale: its translation to Portuguese, cultural adaptation and validation. Rev Lat Am Enfermagem. 2012;20:583-589.

14 Malhotra A, Shah N, Depasquale J, Baddoura W, Spira R, Rector T. Use of Bristol Stool Form Scale to predict the adequacy of bowel preparation - a prospective study. Colorectal Dis. 2016;18:200-204.

15 Gianpiero Manes, Alessandro Repici, Cesare Hassan. Randomized controlled trial comparing efficacy and acceptability of split- and standard-dose sodium picosulfate plus magnesium citrate for bowel cleansing prior to colonoscopy. Endoscopy. 2014;46:662-669.

16 Rex DK. Bowel preparation for colonoscopy: entering an era of increased expectations for efficacy. Clin Gastroenterol Hepatol. 2014;12:458-462.

17 Sharma VK, Chockalingham SK, Ugheoke EA, Kapur A, Ling PH, Vasudeva R, et al. Prospective, randomized, controlled comparison of the use of polyethylene glycol electrolyte lavage solution in four-liter versus two-liter volumes and pretreatment with either magnesium citrate or bisacodyl for colonoscopy preparation. Gastrointest Endosc. 1998;47:167-171.

18 Mayur Brahmania, George Ou, Brian Bressler, Ko HK, Lam E, Telford J, et al. 2 L versus 4 L of PEG3350 + electrolytes for outpatient colonic preparation: a randomized, controlled trial. Gastrointest Endosc. 2014;79:408-416.e4.

19 Lai EJ, Calderwood AH, Doros G, Fix OK, Jacobson BC. The Boston bowel preparation scale: a valid and reliable instrument for colonoscopy-oriented research. Gastrointest Endosc. 2009;69:620-625.

20 Calderwood AH, Jacobson BC. Comprehensive validation of the Boston Bowel Preparation Scale. Gastrointest Endosc. 2010;72:686-692.
