# Supplementary material for: Randomized controlled trial: Standard versus supplemental bowel preparation in patients with Bristol stool form 1 and 2
Source: PLoS One. 2017 Feb 27;12(2):e0171563. doi: 10.1371/journal.pone.0171563 (PMC5328251; doi:10.1371/journal.pone.0171563)
Supplement: S2 Study Protocol — (DOCX) [file pone.0171563.s003.docx]

**研究方案**

**根据Bristol大便性状分型进行个体化肠道准备的前瞻性、随机、对照、单盲、多中心研究**

**一、研究背景和目的**

结肠镜是诊断和治疗结肠疾病的一种标准手段。在结肠镜操作时，充分的肠道准备是对结肠黏膜进行良好观察的必要条件。重要的是，良好的肠道准备可以提高息肉/腺瘤的检出率，避免重复肠镜检查或缩短肠镜随访的时间间隔。失败的肠道准备可导致12-22%的额外费用的增加[1-4]。为了提高肠道准备得总体质量，来自许多国家的内镜医师已经进行了许多的工作和研究[5]。然而，遗憾的是，尽管拥有诸多先进的肠道准备方案，仍然有1/3左右的患者不能达到理想的肠道准备质量[6-9]。

Bristol大便性状分型量表（the Bristol stool form scale, BSFS)是O'Donnell LJD 等人于1988年发明的，目前它已广范应用于胃肠病学研究和临床实践[10-12]。（表一）该量表将大便性状分为7个类型[13]。我们在临床工作中发现，大便性状为Bristol1型和2型的患者在肠镜检查前进行肠道准备时，所获得的肠道准备质量较差，肠道准备失败的比例较其他5种大便类型的患者为高[14]。遗憾的是，目前国内外尚无针对Bristol不同类型对患者进行个体化肠道准备的临床研究[15]。然而，大便性状为Bristol1型和2型患者肠道准备质量差的问题亟待解决[16]。以BSFS为指导的肠道准备有希望成为一种简便可行的新型肠道准备方法。

比沙可啶是一种常用的泻药。有多项研究表明，与4L PEG相比，比沙可啶联合2L方案可获得相似的肠道准备效果[17,18]。

因此，我们评估比沙可啶联合2L PEG-ELP方案对Bristol 1、2型患者肠道准备的质量，并探索一种简单实用的、以Bristol大便性状分型为依据的个体化肠道准备方案，提高接受肠镜检查患者的肠道准备质量，进而更好的服务于临床与科学研究工作。

**二、研究方法**

这是一项前瞻性、随机、对照、单盲、多中心研究。

**1.纳入和排除标准**

**1.1纳入标准：**

大于18周岁预约进行肠镜检查并且能够签署知情同意书的门诊患者。

**2.2排除标准：**

1）结直肠外科手术史；2）严重的结肠狭窄或阻塞性肿瘤；3）炎症性肠病或中毒性巨结肠；4）严重的充血性心力衰竭；5）严重的慢性肾衰竭（肌酐清除率<30ml/min）；6）妊娠期或哺乳期女性；7）不能签署知情同意书者。

**2. 研究单位**

山东大学齐鲁医院、山东省千佛山医院、滨州市人民医院。

**3.分组及干预**

3.1分组:

符号纳入标准的患者自愿签署知情同意书。研究者对患者进行病史采集、记录患者的排便频率并根据患者最近1周的Bristol大便性状分型（见表一、图一）严格按照随机数字表将Bristol大便性状1、2型的患者随机分为A、B两组。随机数字表由计算机生成。采用信封法隐藏随机方案：即为每个入组病例提供唯一的随机信封，研究者拆取信封获知该病例的分组信息。将Bristol大便性状3～7型的患者分入C组。C组的纳入方法为每纳入2名Bristol大便性状1、2型的患者，即纳入1名Bristol大便性状3～7型的患者。

3.2 干预：

A组患者患者应用聚乙二醇电解质散（和爽）2L进行肠道准备；

B组所有患者应用比沙可啶10 mg+聚乙二醇电解质散（和爽）2 L进行肠道准备；

C组所有患者应用聚乙二醇电解质散（和爽）2 L进行肠道准备。

表一：Bristol大便性状分型量表


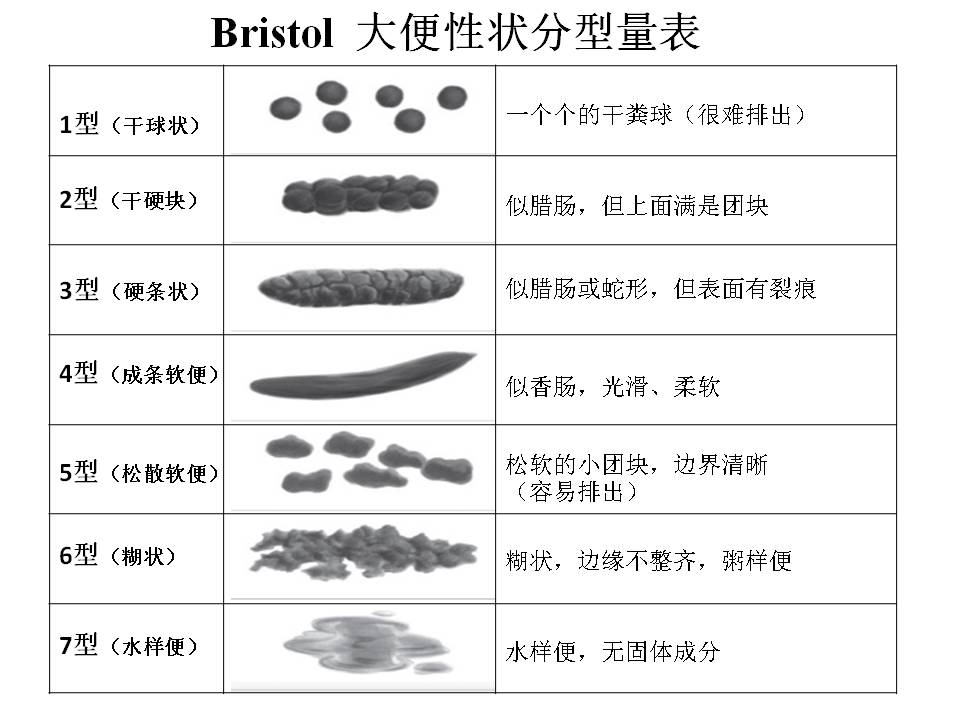


图一 Bristol大便性状分型量表

3.3肠道准备方法:

患者于肠镜检查的前一天早餐、午餐应为低渣饮食，晚餐应该为清流质饮食。

A、C组患者于肠镜检查前7小时服用和爽1L，于肠镜检查前4小时再次服用和爽1L。B组患者于肠镜检查前一天晚上20点口服比沙可啶10 mg（2片）；于肠镜检查前7小时服用和爽1L，于肠镜检查前4小时再次服用和爽1L。

肠镜检查于下午13点30分至17点之间进行。泻药服用速度为每半小时500ml。鼓励患者在服完泻药之后多喝清水。在结肠镜检查前2小时应停止饮水。

3.4患者教育

在预约肠镜时，由一名护士向患者说明肠道准备的方法及注意事项，并且向患者发放一份印有肠道准备方法及注意事项的卡片，以便患者进行正确的肠道准备。

4.评分标准

本实验应用Boston肠道准备评分系统（the Boston Bowel Preparation Scale, BBPS）评估肠道准备的效果。

BBPS具体为：

在进行肠镜检查时，研究者（评估肠道准备质量者）分别对右侧结肠（包括盲肠和升结肠）、横结肠（包括肝区和脾区）及左侧结肠（包括降结肠、乙状结肠和直肠）3个肠段的肠道准备情况按4级（0~3分）评分系统进行评分。肠道准备评分标准如下：

0，肠道准备质量极差，肠段内有固体粪便存留，无法看到结肠黏膜。

1，可看到部分结肠黏膜，但是由于污斑、残留大便 、和/或不透明液体致使其他区域的的结肠黏膜不能被很好地观察。

2，存有少量的污斑、大便 和/或不透明液体，但可清楚地观察到结肠黏膜。

3，结肠黏膜清晰可见，并且无污斑、大便 和/或不透明液体存在。

每个肠段所得评分相加，即为总的肠道准备的Boston评分（0~9分）。如果内镜医师因为肠道准备不充分而停止肠镜检查，那么所有未观察到的肠段的评分记为0分。肠道准备质量分为“充分(adequate)”（每个肠段得分均≥2分）和“不充分（inadequate）”（一个或多个肠段得分＜2分）。

在研究开始进行之前，各分中心的研究者（（评估肠道准备质量者））预先按照Boston评分系统对10例患者的肠道准备情况进行评分，目的在于熟悉并准确应用Boston评分系统。

5.评价指标及评估方法

5.1 主要评价指标：肠镜检查时，三组患者肠道准备的Boston评分是否有统计学差异[19-20]。

5.2 次要评价指标包括：患者的依从性、耐受性、睡眠质量、不良反应、结肠镜检查成功率、到达盲肠时间、退镜时间、息肉检出率、非息肉病变检出率及内镜医师建议重新进行肠道准备率等。

在结肠镜检查前，每个研究单位分别由一名护士就患者对肠道准备的依从性（**compliance**）、耐受性（**tolerance**）、可接受性（**acceptability**）以及患者再次接受相同方案肠道准备的意愿等进行评估。评估方法以问卷形式进行（见附表）。依从性评估内容包括开始服用泻药的时间是否正确、是否按医嘱限制饮食、是否按医嘱服用泻药；耐受性包括睡眠质量、不良反应以及患者所接受肠道准备方案的满意度，其中应用Likert评分法对患者的不良反应程度及满意度进行评估（0分代表非常满意；7分代表完全无法忍受）。同样，应用Likert评分法就患者所喝完规定剂量泻药的难度进行评估（0分代表非常轻松；7分代表完非常困难）。

每个研究单位分别由一名研究者对每位患者的肠道准备质量（Boston肠道准备评分）、到达盲肠时间、退镜时间、结肠镜下所见、内镜医师是否建议重新进行肠道准备以及患者是否愿意再次相同方法的肠道准备进行评估并记录。

以上研究者和内镜医师对患者大便 的Bristol分型及肠道准备方法被设盲。

**6.伦理审批**

各研究单位均进行并通过伦理委员会审批。

研究设计、执行、分析等各环节遵循国内外各种伦理要求和规范。

**7.样本量估计**

本试验的主要终点为患者肠道准备的充分率（**adequate rate**）（将患者肠道准备质量按BBPS评估每个肠段得分均≥2分）。在本研究开始前，对试验的样本量进行估计。假定按两种方案进行肠道准备的充分率相差为15%。A组肠道准备方案所达到的充分率约为60%。设α=0.05，β=0.1，双侧检验，我们通过计算本研究每组所需的样本量为至少203例。假设失访率为10%，因此每组至少需要224例患者。总共需要病例不少于672例，本研究计划纳入700例患者。本研究的次要终点为各组中息肉性病变和非息肉性病变的检出率、患者的依从性及不良反应的发生率。

各中心采用竞争入组法（competitive enrollment），每个研究分中心首次分配A、B组1个block（50例），C组25例。纳入患者越快的中心所分配的样本数越多。

**8.统计方法**

意向性（Intention-to-treat, ITT）分析用于评估主要终点和结肠镜发现。应用卡方检验或Fisher确切检验分析分类变量；连续型变量用均数与标准差表示，应用Student t检验进行分析。*p*＜0.05为差异有统计学意义。采用SPSS分析统计软件进行数据分析（version 18，SPSS Inc.，Chicago，IL，USA）。
